# Transition-Metal-Catalyzed Directed C–H Bond Functionalization with Iodonium Ylides: A Review of the Last 5 Years

**DOI:** 10.3390/molecules29153567

**Published:** 2024-07-29

**Authors:** Juting Liao, Dulin Kong, Xiaoyang Gao, Ruirui Zhai, Xun Chen, Shuojin Wang

**Affiliations:** Engineering Research Center of Tropical Medicine Innovation and Transformation of Ministry of Education, International Joint Research Center of Human-Machine Intelligent Collaborative for Tumor Precision Diagnosis and Treatment of Hainan Province, Hainan Provincial Key Laboratory of Research and Development on Tropical Herbs, School of Pharmacy, Hainan Medical University, Haikou 571199, China

**Keywords:** iodonium ylides, transition metal-catalyzed, C–H activation

## Abstract

Transition-metal-catalyzed directed C–H functionalization with various carbene precursors has been widely employed for constructing a wide range of complex and diverse active molecules through metal carbene migratory insertion processes. Among various carbene precursors, iodonium ylides serve as a novel and emerging carbene precursor with features including easy accessibility, thermal stability and high activity, which have attracted great attention from organic chemists and have achieved tremendous success in organic transformation. In this review, recent progress on the application of iodonium ylides with multifunctional coupling characteristics in C–H bond activation reactions is summarized, and the potential of iodonium ylides is discussed.

## 1. Introduction

Metal carbenoid species resulting from carbene precursors and different metals have been widely recognized as an important and versatile active intermediate to build complex and diverse skeletons in organic synthesis [1,2,3]. Carbene precursors, such as diazo compounds, enynones, hydrazones, vinylene carbonates, sulfoxonium ylides, and others, have been extensively explored as coupling partners in various organic reactions [4,5]. Despite this great progress, it is greatly demanding and challenging to continuously seek an efficient and novel carbene precursor as a substitute. Iodonium ylides, which exhibit good thermal stability, easy accessibility and high reactivity, are known to construct various structures, especially cyclic ones [6,7]. In the past decade, iodonium ylides have been mainly used as a carbene-transfer reagent and as a carbenoid insertion into X–H (X = O, S, N) bonds of nucleophiles for constructing the C–X bond [8,9].

**Scheme 1:**
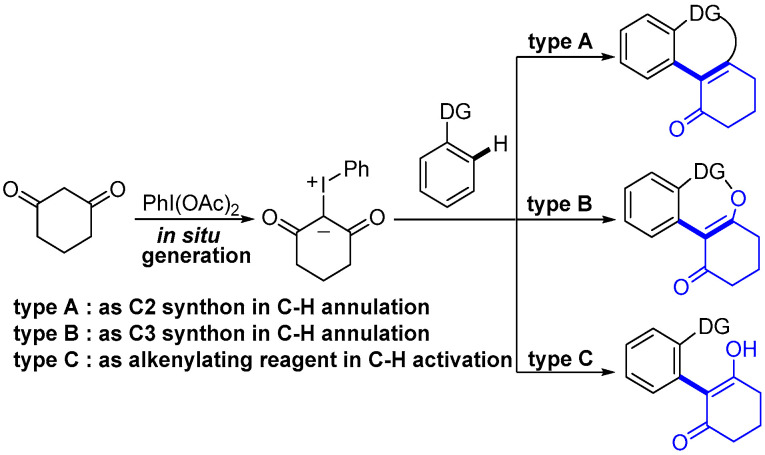
Transition–metal–catalyzed C–H bond activation reaction with iodonium ylides.

Transition-metal-catalyzed directed C–H activation has emerged as an atom/step-economical method for the rapid synthesis of various useful molecules [10,11,12,13]. In contrast to the traditional cross-coupling of iodonium ylides with nucleophiles, directly using iodonium ylides as a significant coupling partner in transition-metal-catalyzed C–H activation reactions are especially appealing. In 2020, the Li group first applied iodonium ylides as carbene precursors in Rh(III)-catalyzed directed C–H activation reactions under mild and redox-neutral conditions [14]. Since then, a large number of studies have reported employing iodonium ylides as a novel and emerging carbene precursor in transition-metal-catalyzed chelation-assisted C–H functionalization [15,16,17,18,19,20,21,22,23,24,25,26,27,28,29,30,31,32,33,34,35,36,37,38,39,40,41,42,43,44,45,46,47,48,49,50,51,52,53,54]. To the best of our knowledge, only one simple review of 21 examples using iodonium ylides in transition-metal-catalyzed C–H functionalization was reported by the Kanchupalli group in 2022 [55]. However, after the publication of Kanchupalli’s review, there are many studies (about 20 references) on the C–H functionalization of iodonium ylides, which was intensively reported in 2023 and 2024. Herein, we aim to provide a comprehensive overview of transition-metal-catalyzed C–H functionalization utilizing iodonium ylides with multifunctional coupling characteristics over the last 5 years. It should be noted that most of the carbene precursors were limited to the cyclic 1,3-diketones-derived iodonium ylides, because this type of transformation may be sensitive to the steric hindrance of iodonium ylides. Based on the different forms of iodonium ylides in C–H transformations, this review article is organized into three sections: (1) those serving as C2 synthon in C–H annulation, (2) serving as C3 synthon in C–H annulation, or (3) serving as an alkenylating reagent in C–H functionalization (Scheme 1).

## 2. Iodonium Ylides Serve as C2 Synthon in C–H Annulation

C2 synthon is one of the most common synthetic forms in the C–H activation reaction, which could be readily converted into the valuable heteroaromatic moieties by undergoing [n+2] annulation with the C–H bond. The utilization of iodonium ylides as C2 synthon are extensively studied in transition-metal-catalyzed C–H activation reactions.

### 2.1. Amide Group-Directed C–H Annulation

**Scheme 2:**
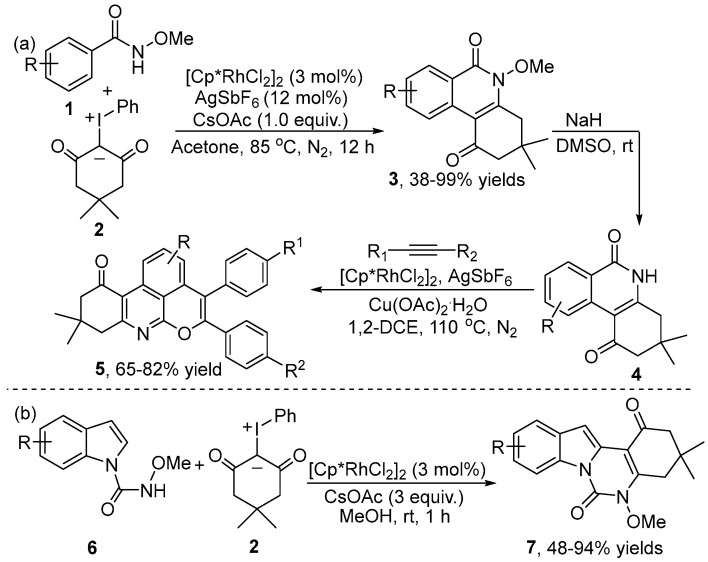
Rh(III)–catalyzed [4+2] annulation of (**a**) *N*-methoxyarylzamides and (**b**) *N*-carboxamide indole with iodonium ylides.

In 2020, Mayakrishnan and co-workers [15] reported Rh(III)-catalyzed arene C–H annulation of *N*-methoxybenzamides **1** with iodonium ylide **2** as a carbene precursor for the assembly of dihydrophenanthridines **3** (Scheme 2a). A wide range of substrates were compatible with this catalytic system. Importantly, the potential synthetic utility of the coupling product **3** was demonstrated by synthesizing pyranoisocoumarin **5**. The optoelectronic properties of product **5** were tested by UV/vis and fluorescence spectrometers. The results showed excellent optical properties with emission maxima, with the potential to be used in the photoelectric and biomedical imaging fields. Very shortly after Mayakrishnan’s publication, Ji and co-workers [16] also developed a similar procedure (Scheme 2b). They disclosed a Rh(III)-catalyzed cross-coupling reaction of *N*-carboxamide indole **6** with iodonium ylide **2** to deliver indoloquinazolinone **7**. Various substrates with different substituents were well tolerated in this transformation.

**Scheme 3:**
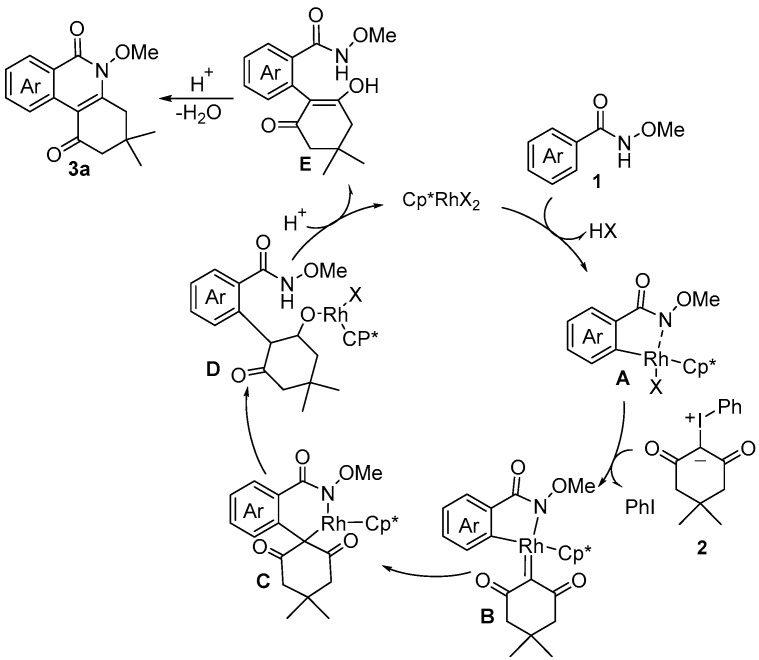
Proposed reaction mechanism for the [4+2] annulation of *N*-methoxyarylzamides with iodonium ylides.

A plausible mechanism is provided in Scheme 3. After the coordination of *N*-methoxybenzamide **1** to a cationic Rh(III) catalyst, C–H bond activation occurred and gave five-membered cyclometalated species **A**. Subsequently, the coordination of iodonium ylide **2** and the elimination of PhI led to the formation of Rh(III) intermediate **B**, which underwent the migratory insertion and protonation process to yield intermediate **E**. Finally, the intramolecular nucleophilic addition and dehydration annulation process generated the desired product **3a.**

**Scheme 4:**
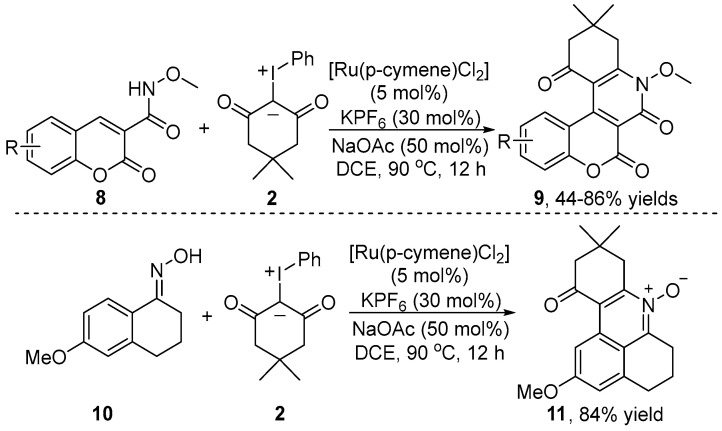
Ru(II)–catalyzed C–H [4+2] annulation of coumarins and oxime-derivatized tetralone with iodonium ylides.

In 2023, Asish and co-workers [17] also demonstrated the assembly of coumarin-3-carboxamide **9** via [4+2] cyclization of coumarin **8** with iodonium ylide **2** using an *N*-methoxy carboxamide unit as the chelating fragment (Scheme 4). Moreover, when oxime-derivatized tetralone **10** was used as an arene substrate under similar reaction conditions, the corresponding tetracyclic pyridine-*N*-oxide product **11** was obtained in good to excellent yields. A variety of substrates bearing various functional groups were well tolerated under mild and redox-neutral conditions, in which 30 mol% KPF_6_ could absolutely substitute the more expensive silver salts. Significantly, pyridine-*N*-oxide product **11** showed excellent fluorescence quenching activity, which could be applied to a fluorescence sensing probe.

**Scheme 5:**
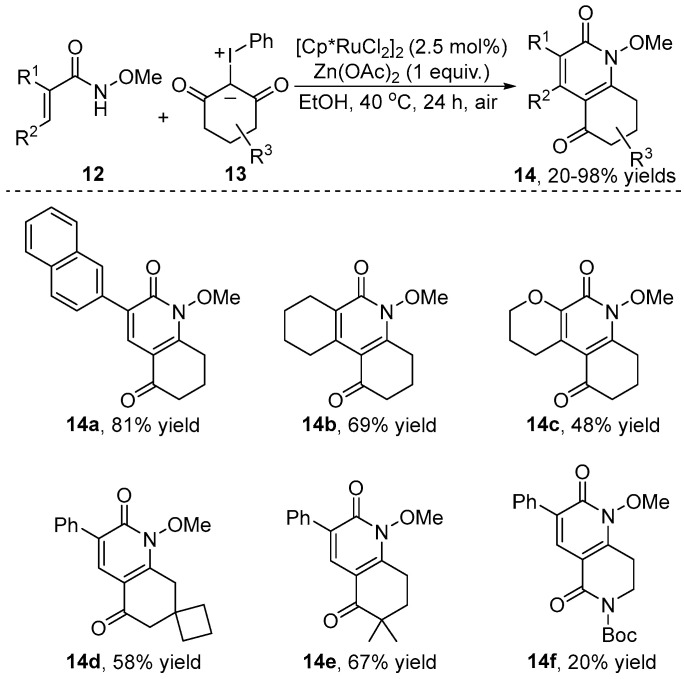
Rh(III)–catalyzed [4+2] annulation of *N*-arylpyrazolidinones with iodonium ylides.

Subsequently, the Wu group [18] also accomplished the Rh(III)-catalyzed olefinic [4+2] cyclization of *α, β*-unsaturated amide **12** with iodonium ylide **13** for the synthesis of dihydroquinoline-2,5-dione **14** in moderate to high yields (Scheme 5). The transformation featured water and air compatibility and excellent functional group tolerance. Interestingly, the authors applied this protocol to a large-scale reaction with a low catalyst load (0.25–1.5 mol%) and extracted the Rh catalyst three times during the [4+2] cyclization at a 65% yield.

### 2.2. Hydrazine Group-Directed C–H Annulation

**Scheme 6:**
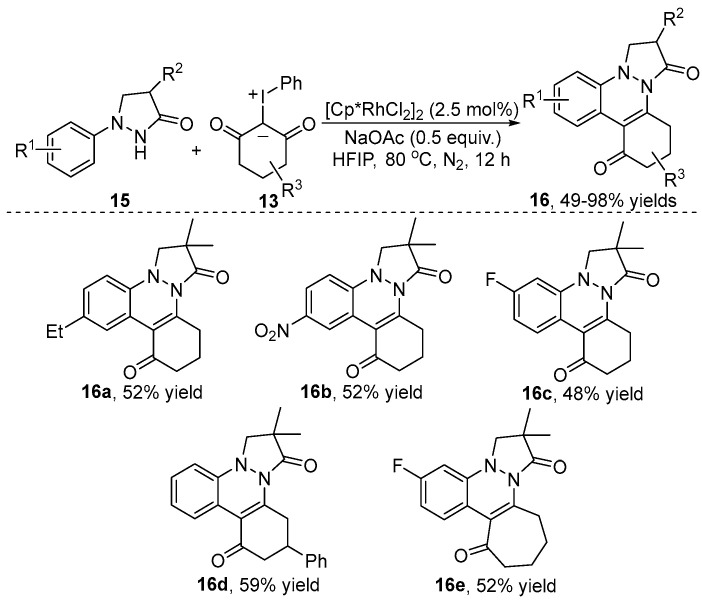
Rh(III)–catalyzed [4+2] annulation between pyrazolidinones and iodonium ylides.

In 2022, Li and co-workers [19] described a Rh(III)-catalyzed C–H coupling/cyclization of pyrazolidinone **15** with iodonium ylide **13** for the synthesis of pyrazolo[1,2-*a*]cinnoline **16** (Scheme 6). This transformation proceeded under oxidant-free conditions and resulted in a decent to high yield. An excellent functional group compatibility was observed in this reaction. The H/D exchange experiment suggested a reversible C–H activation process.

**Scheme 7:**
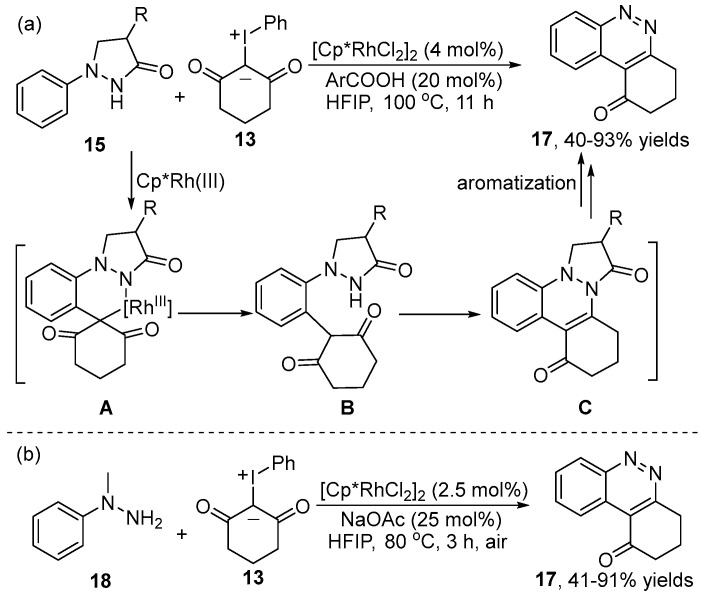
Rh(III)–catalyzed [4+2] annulation for the synthesis of cinnoline.

Owing to the valuable applications of cinnoline in the medicinal and organic areas, a Rh(III)-catalyzed [4+2] annulation for the synthesis of cinnoline **17** had been realized by Hu’s group [20] in 2022 (Scheme 7a). Compared with the above-mentioned mechanism using the same substrates, the [4+2] cyclization intermediate **C** was further aromatized under HFIP to give annelated cinnoline **17**. In this same time, Yu and co-workers [21] also explored the construction of cinnoline **17** from *N*-methyl arylhydrazine **18** and iodonium ylide **13** (Scheme 7b). A large number of functional groups were compatible in this Rh(III)-catalyzed C–H activation reaction. Similarly, product **17** was obtained through a C–H activation, dehydration and demethylative aromatization process.

**Scheme 8:**
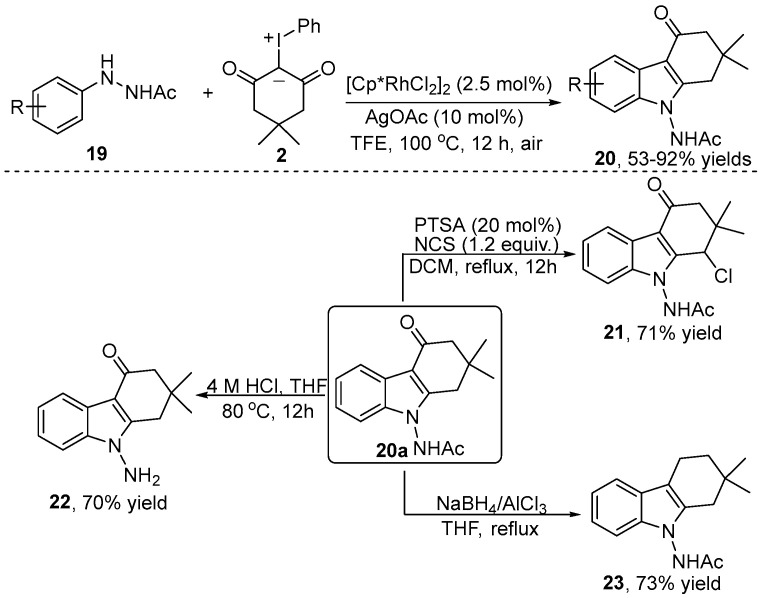
Rh(III)–catalyzed [3+2] annulation of arylhydrazines with iodonium ylides.

Tetrahydrocarbazol-4-ones are a privileged scaffold found in numerous bioactive molecules and drugs, such as ondansetron and heat shock protein inhibitor. In this regard, Liu et al. [22] independently reported an approach to produce tetrahydrocarbazol-4-one **20** via the [4+2] annulation of readily available arylhydrazine **19** with iodonium ylide **2** by using [Cp*RhCl_2_]_2_ as a catalyst and AgOAc as an additive (Scheme 8). A variety of substrates with different substituents were well tolerated in this transformation. It should be noted that the authors demonstrated the utility of this strategy by performing a gram-scale experiment of product **20**. The tetrahydrocarbazol-4-one **20a** was further derived to produce the corresponding compounds **21, 22** and **23** via substitution, hydrolysis and reductive hydrogenation reactions, respectively.

### 2.3. Sulfamide Group-Directed C–H Annulation

**Scheme 9:**
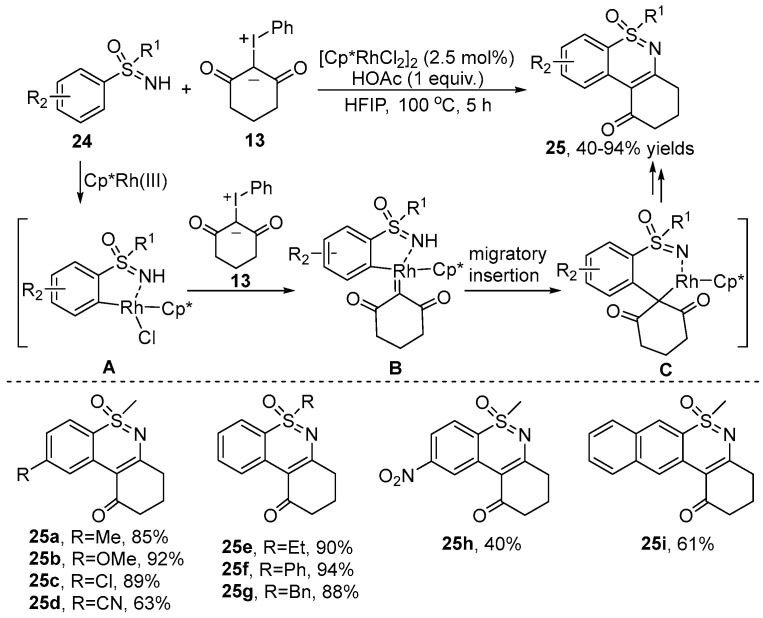
Rh(III)–catalyzed [4+2] annulation of *S*-aryl-sulfoximines with iodonium ylides.

In 2021, Pan’s group [23] demonstrated a Rh(III)-catalyzed C–H and N–H bond functionalization of *S*-aryl-sulfoximine **24** with iodonium ylide **13** by using the sulfoximine moiety as a directing group (Scheme 9). This transformation provided a new way to access multifariously switched tricyclic and tetracyclic 1,2-benzothiazines **25** in moderate to good yields. Various substrates with different substituents were well tolerated in this transformation. In the case of *S*-2-naphthalenyl sulfoximine, the reaction predominantly occurred at the C–H bond with less steric hindrance to generate the single product **25i**. To explore the reaction mechanism, a stable cyclometalated Rh(III) complex **A** was isolated, and it was found to efficiently catalyze the reaction to provide the desired product.

**Scheme 10:**
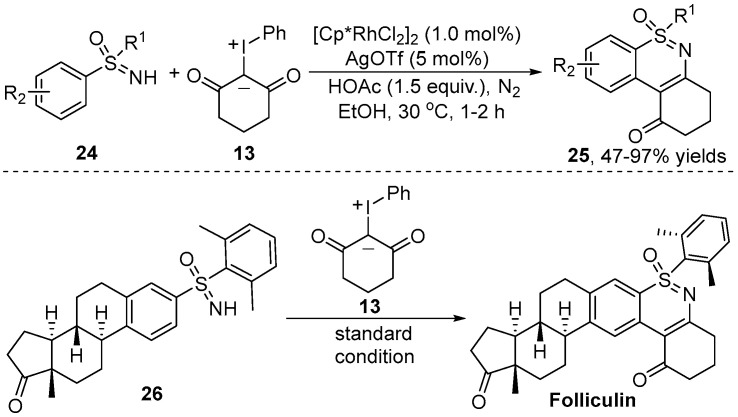
Rh(III)–catalyzed [4+2] annulation for the synthesis of a Folliculin analog.

More recently, similar work was reported independently by Chen and co-workers [24] (Scheme 10). In contrast with Pan’s work, this transformation was performed under low temperature conditions. In addition, this reaction was characterized by using EtOH as a green solvent under oxygen/water-insensitive conditions and with the requirement for only a low catalyst load. To demonstrate the potential application of this annulation reaction, the authors used this methodology to rapidly synthesize a complex pharmaceutical Folliculin analog.

### 2.4. Nitroso Group-Directed C–H Annulation

**Scheme 11:**
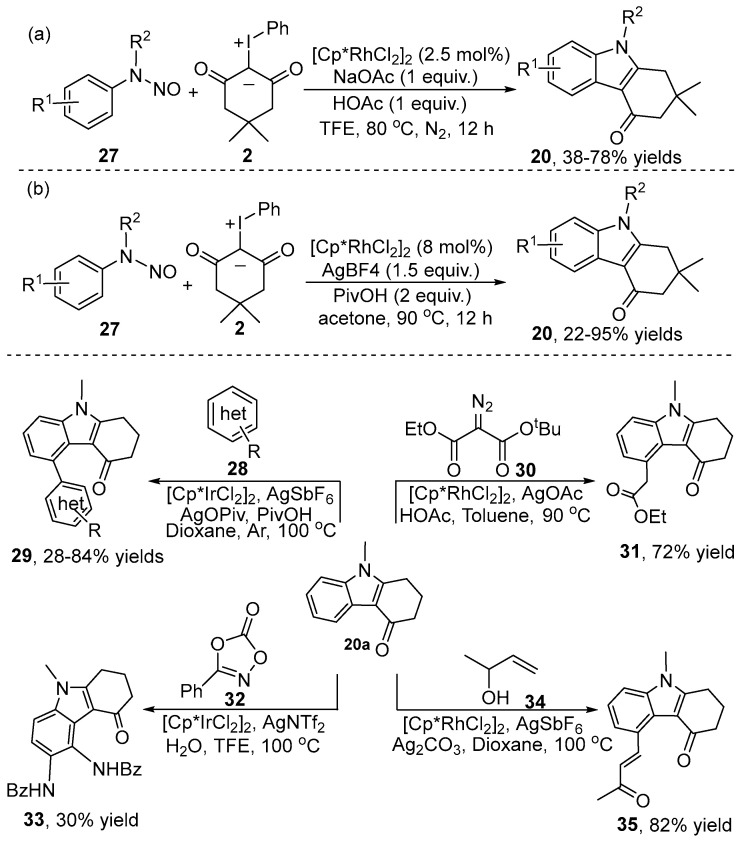
Rh(III)–catalyzed [3+2] cyclization of *N*-nitrosoanilines with iodonium ylides.

The nitroso group has been recognized as an efficient directing group and an internal oxidant in various C–H bond redox-neutral functionalization/annulation reactions. In recent years, a Rh(III)-catalyzed C–H bond [3+2] cyclization approach was applied to form tetrahydrocarbazol-4-one **20** using a nitroso motif as the directing group, by the Yang group [25] (Scheme 11a). The reaction conditions were mild without any additional oxidant, and a variety of *N*-nitrosoanilines and iodonium ylides were compatible. At almost the same time, a similar Rh(III)-catalyzed synthesis of polysubstituted tetrahydrocarbazol-4-one **20** employing the same substrates was developed by Liu and co-workers [26] (Scheme 11b). The authors further demonstrated metal-catalyzed C–H bond activation reactions at the C5-position of tetralydrocarbzol-4-one **20** using different coupling partners under mild conditions, including alkylation, alkenylation, amidation and (hetero)arylation.

**Scheme 12:**
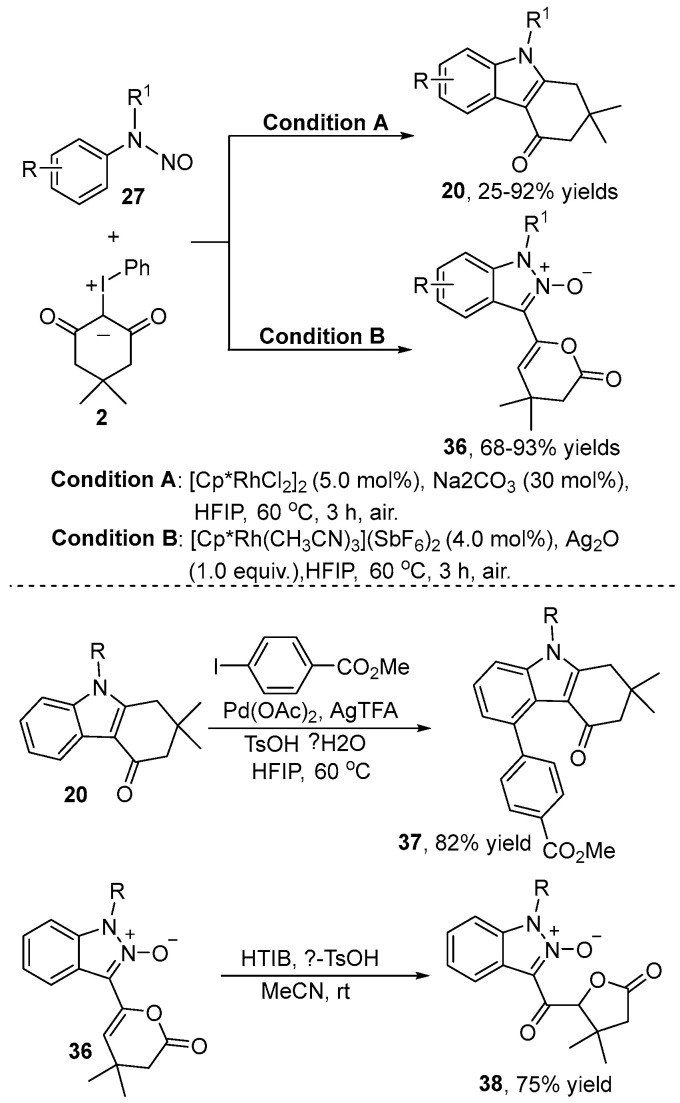
Condition-controlled Rh(III)–catalyzed C–H annulation of *N*-nitrosoanilines and iodonium ylides.

Very shortly after Liu’s publication, the Fan group [27] also realized a condition-controlled C–H bond functionalization of *N*-nitrosoaniline **27** with iodonium ylide **2** (Scheme 12). This reaction was conducted in HFIP using [Cp*RhCl_2_]_2_ as the catalyst in the presence of Na_2_CO_3_ at 60 °C to produce tetrahydrocarbazol-4-one **20** by selectively controlling the [3+2] annulation. In contrast, switching the catalyst to [RhCp*(MeCN)_3_](SbF_6_)_2_ and the additive to Ag_2_O resulted in the formation of pyranone-tethered indazole **36** via [4+1] annulation. The compound **37** and the γ-butyrolactone derivative **38** were produced by a regioselective C–H alkylation and a ring contraction reaction, respectively, which proved the successful application of the catalytic system.

**Scheme 13:**
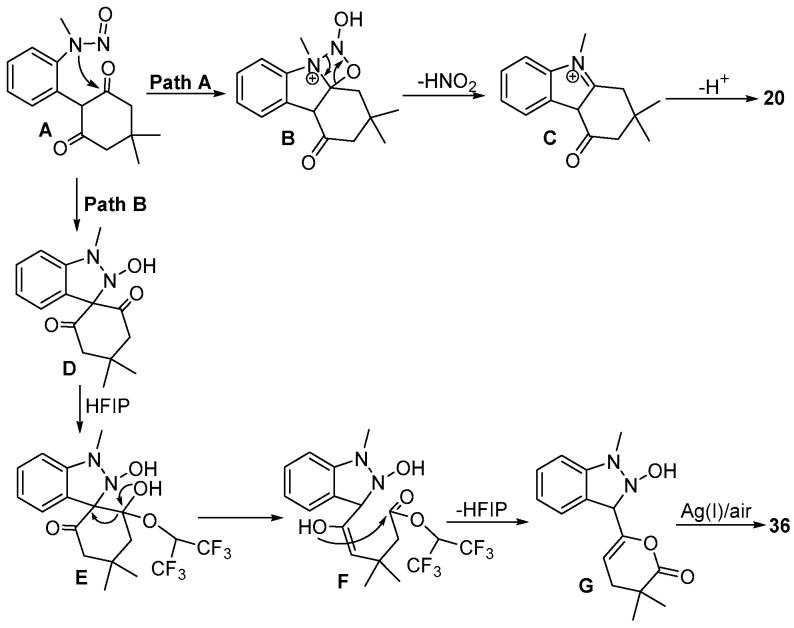
Proposed reaction mechanism for condition-controlled Rh(III)–catalyzed C–H annulation of *N*-nitrosoanilines and iodonium ylides.

This transformation underwent C–H bond activation, carbene migratory insertion and protonation processes to deliver alkylation intermediate **A**. In pathway **A**, the intramolecular cyclization and denitrosation of intermediate **A** provided the desired product **20**. However, in pathway **B**, the intermediate **D** formed via intramolecular C-nucleophilic addition underwent ring opening of cyclohexanedione to generate the intermediate **E**. Intermediate **E** underwent tautomerization and intramolecular transesterification/cyclization to furnish intermediate **G**, which finally delivered product **36** via Ag(I)/O_2_-mediated aromatization-driven oxidative dehydrogenation (Scheme 13).

**Scheme 14:**
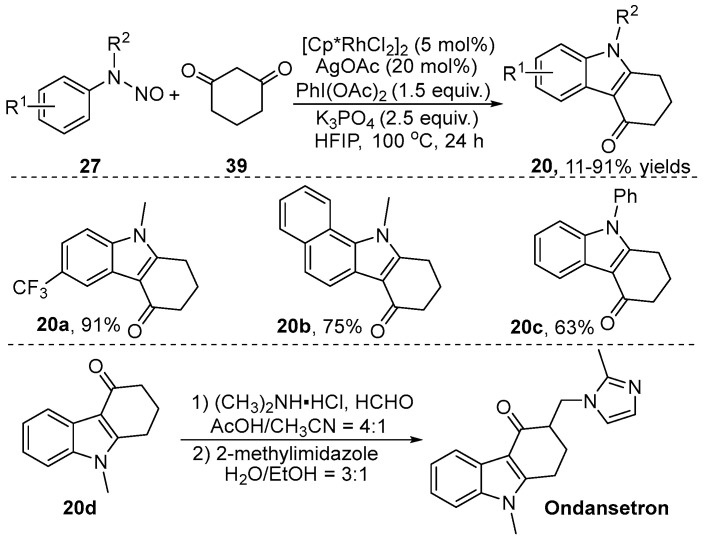
Rh(III)–catalyzed [3+2] annulation of *N*-nitrosoanilines with cyclohexane-1,3-dione.

From the perspective of step economy, the in situ generation of iodonium ylides as carbene precursors in a one-pot manner is very attractive. At almost the same time, our group [28] explored a Rh(III)-catalyzed direct C–H bond tandem [4+2] annulation of *N*-nitrosoaniline **27** by using simple cyclohexane-1,3-dione **39** as a coupling partner through two C–H bond cleavages, producing tetrahydrocarbazol-4-one **20**, and in which the in situ generation of iodonium ylides was achieved (Scheme 14). Subsequently, the biological application of these target coupling products was investigated. The results showed that they displayed significant cytotoxicity against HepG_2_ cells. Furthermore, product **20d** could be further functionalized to assemble ondansetron using the Mannich reaction.

### 2.5. Amino Group-Directed C–H Annulation

**Scheme 15:**
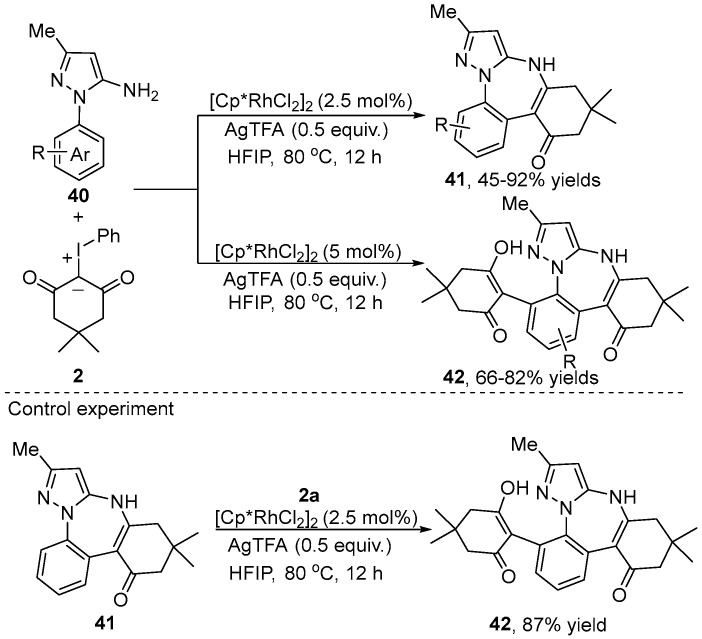
Rh(III)–catalyzed aromatic NH_2_ moiety-directed [5+2] annulation of 1-aryl-5-aminopyrazoles with iodonium ylides.

In 2023, Huang’s group [29] accomplished a Rh(III)-catalyzed selective mono- and dual-C–H bond unsymmetrical functionalization/cyclization of 1-aryl-5-aminopyrazole **40** and iodonium ylide **2** using the aromatic NH_2_ moiety as the directing group, leading to fused benzodiazepine skeletons **41** and **42** (Scheme 15). The amount of iodonium ylides played an important role in chemo-selectivity. In particular, dual-C–H functionalization proceeded via the utilization of three equivalence of iodonium ylides to give product **42**. To elucidate the reaction mechanism, the reaction between product **41** and iodonium ylides was performed under optimal reaction conditions to obtain an 87% yield of product **42.** This result indicated that product **41** was a possible intermediate in the dual C–H functionalization process.

**Scheme 16:**
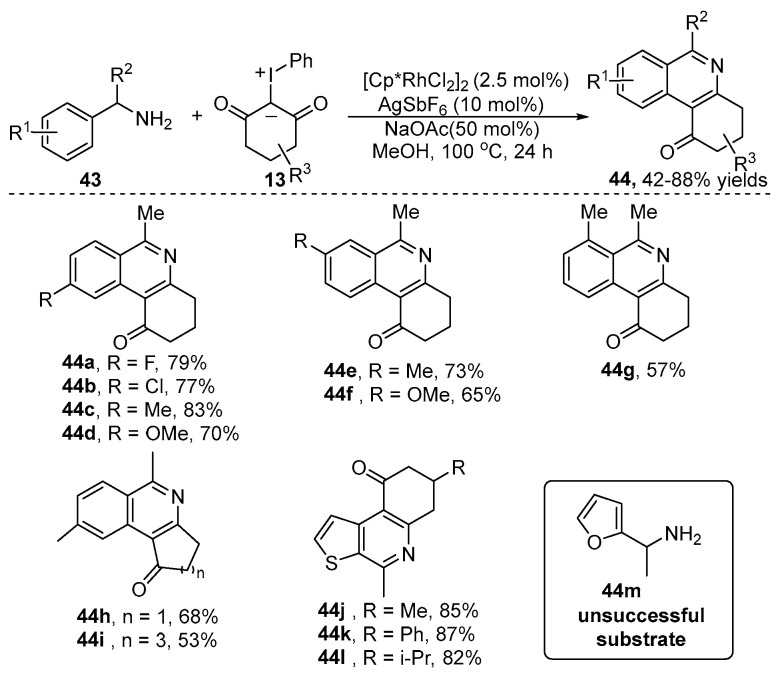
Rh(III)–catalyzed aliphatic NH_2_ group-directed [4+2] annulation of primary benzylamines with iodonium ylides.

Compared with the aromatic NH_2_ group, the aliphatic NH_2_ group-directed C–H functionalization has attracted great attention because the aliphatic NH_2_ unit has poor coordination abilities and is easily oxidized by oxidants. In this aspect, our group [30] revealed a Rh(III)-catalyzed free aliphatic amines-assisted arene C–H bond coupling/annulation of primary benzylamine **43** with iodonium ylide **13** using air as a green oxidant (Scheme 16). This reaction provided a general, green and step-economic approach to construct a wide range of dihydrophenanthridin-1-one **44**, which are bioactive scaffolds found in numerous natural products. Excellent functional group tolerance and regioselectivity of this catalytic system were observed, except for the furanylmethanamine (**44m**).

**Scheme 17:**
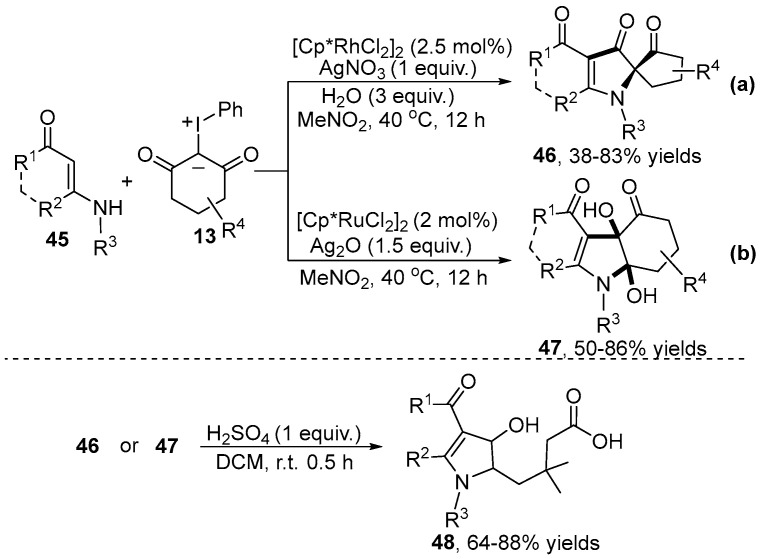
Transition–meta–catalyzed C–H cyclization of enaminones with iodonium ylides.

Except for the aromatic C–H bond, the olefinic C–H bond functionalization/cyclization using iodonium ylides as carbene precursors was recently reported by the Yu group [31,32] (Scheme 17). This protocol involved a novel Rh(III)-catalyzed cascade alkenyl C–H activation and a subsequent pinacol rearrangement reaction of enaminone **45** with iodonium ylide **13**, producing a series of 2-spirocyclo-pyrrol-3-ones **46** in good to high yields (Scheme 17a) [31]. However, a range of dihydroxy hexahydro-4H-indol-4-ones **47** were obtained when using Ru(II) as a catalyst (Scheme 17b) [32]. Moreover, these *N*-heterocyclic products could all be further functionalized to product **48** via the open-ring/hydrolysis of the spiro ring under H_2_SO_4_-mediated conditions. Notably, an ^18^O labeling experiment indicated that water served as the oxygen source for the newly generated carbonyl group in this reaction system.

**Scheme 18:**
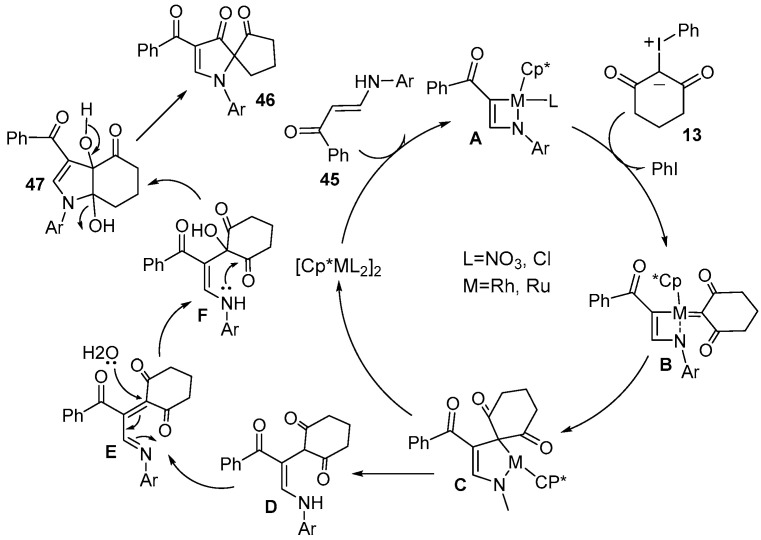
Proposed reaction mechanism for the cyclization of enaminone with iodonium ylides.

A plausible mechanism was proposed (Scheme 18). After the coordination of enaminone **45** to a cationic metal catalyst, direct alkenyl C–H bond activation occurred and gave four-membered cyclometalated species **A**. Subsequently, coordination of iodonium ylide **13** and the elimination of PhI led to the formation of metal intermediate **B**, which was followed by the migratory insertion and protonation processes to generate intermediate **D**. Then, the intermediate **E** obtained through the oxidative dehydrogenation of intermediate **D** underwent intermolecular nucleophilic addition and intramolecular nucleophilic cyclization to generate product **47**. Product **47** could be further converted to **46** by the dehydration, annulation and pinacol rearrangement.

**Scheme 19:**
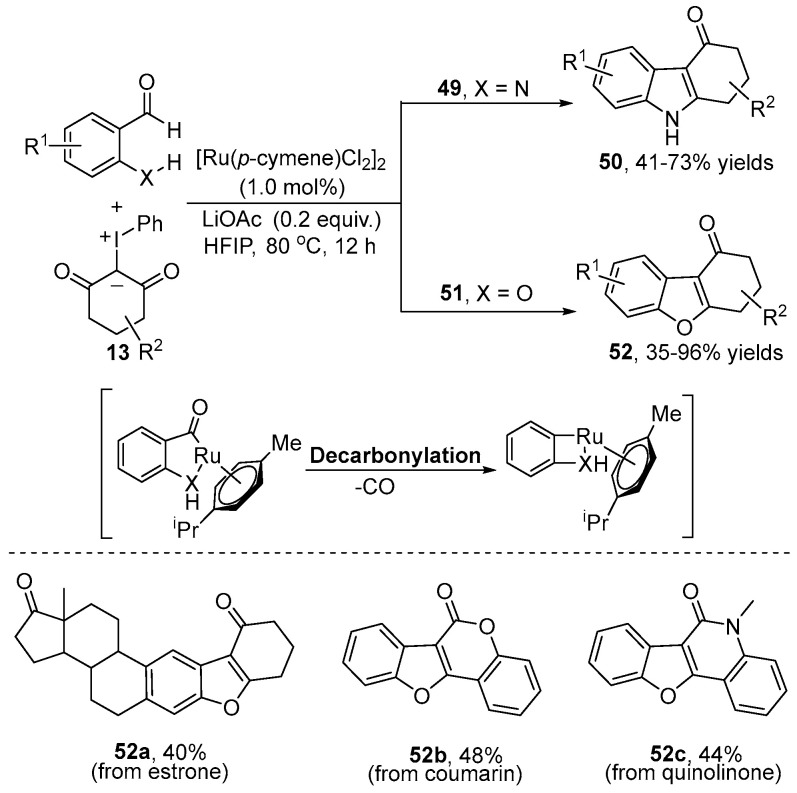
Ru(II)–catalyzed aldehydic C–H [3+2] annulation with iodonium ylides.

Apart from the C–H bonds with arenes and olefins, the NH_2_-directed aldehydic C–H functionalization with iodonium ylides was explored by the Song group [33] in 2022 (Scheme 19). They selected 2-aminobenzaldehyde **49** as a substrate to react with iodonium ylide **13** in the presence of the inexpensive Ru(II) catalyst, giving NH-free carbazolone **50** in moderate to excellent yields via decarbonylative alkylation and annulation processes. Interesting, hydroxy group-directed C–H functionalization with iodonium ylides is also compatible with this catalytic system. The mechanism was proposed to involve aldehydic C–H activation, decarbonylation, coordination, migratory insertion, protonolysis, nucleophilic attack and dehydration processes.

### 2.6. Heterocyclic Group-Directed C–H Annulation

**Scheme 20:**
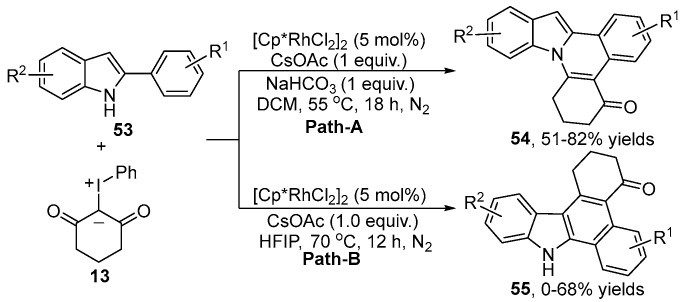
Condition-controlled Rh(III)–catalyzed [4+2] annulation of 2-arylindoles with iodonium ylides.

In view of the biological and pharmacological activities of the indolo [2,1-*a*]isoquinoline derivatives and benzo[*a*]carbazoles, Kanchupalli [34] developed a condition-controlled Rh(III)-catalyzed regioselective C–H activation of 2-arylindole **53** with iodonium ylide **13**, leading to the generation of indolo [2,1-*a*]isoquinolines derivative **54** and benzo[*a*]carbazole **55** (Scheme 20). This protocol was conducted in DCM using NaHCO_3_ as a base to merely produce the indolo [2,1-*a*]isoquinolines derivative **54**. In contrast, switching DCM to HFIP without any base led to benzo[*a*]carbazole **55** with an excellent chemoselectivity. The H/D exchange experiment and KIE experiment revealed that the C–H activation process was reversible and was not involved in the rate-determining step.

**Scheme 21:**
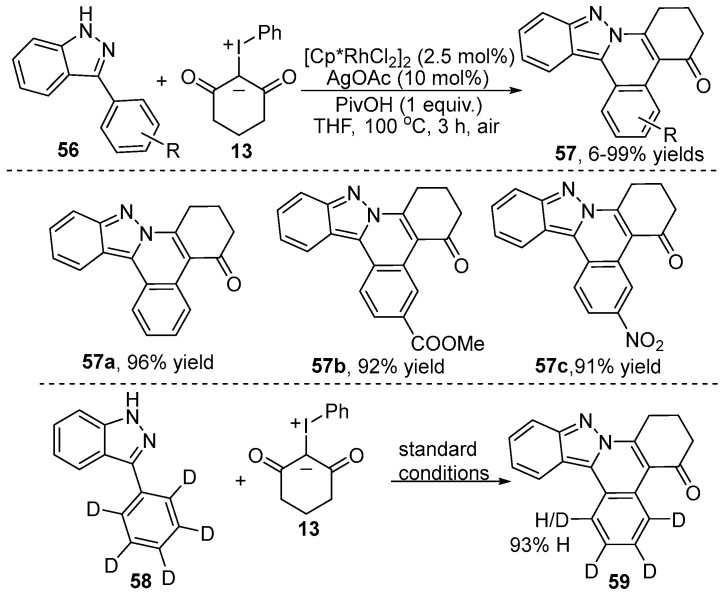
Rh(III)–catalyzed [4+2] annulation of 3-aryl-1H-indazoles and iodonium ylides.

More recently, a similar strategy was reported independently by Cui and co-workers [35] (Scheme 21). They employed the imidazole group as a directing group to realize a Rh(III)-catalyzed C–H alkylation and a subsequent intramolecular [4+2] annulation of 3-aryl-1*H*-indazole **56** with iodonium ylide **13**. This protocol offered a rapid route to a range of tetracyclic and pentacyclic aza-heterocyclics **57**, which could be transformed into a fluorescent dye and biofluorescent probes. Additionally, the H/D exchange experiment suggested a reversible C–H activation process.

**Scheme 22:**
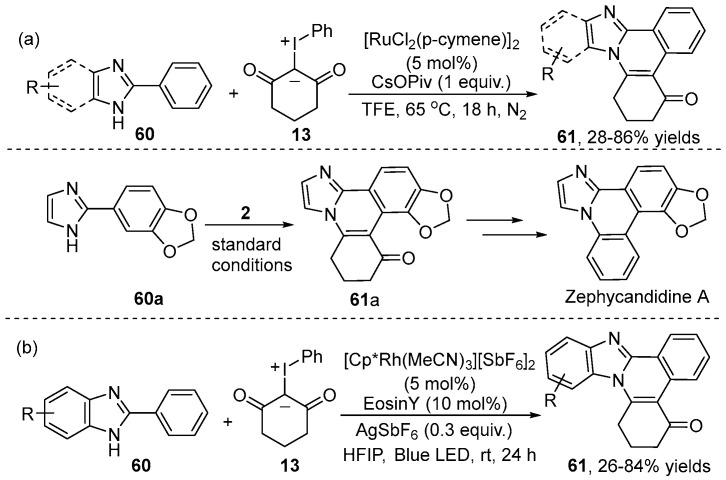
Ru(II)–catalyzed [4+2] cyclization of 2-arylbenzimidazoles with iodonium ylides.

Recently, Ru(II) catalyst has received great attention in C–H functionalization due to advantages like higher catalytic activity, excellent regioselectivity and low cost. In this regard, the group of Kanchupalli developed a Ru(II)-catalyzed [4+2] annulation of 2-arylbenzimidazole **60** with iodonium ylide **13** (Scheme 22a) [36]. This protocol provided easy access to a wide variety of useful substituted tetracyclic and pentacyclic bridgehead *N*-heterocycles **61**, which had been utilized to synthesize the core moiety of the natural product zephycandidine **A** with significant anti-tumor and anti-acetylcholinesterase activities. At almost the same time, Wu and co-workers [37] also reported similar work (Scheme 22b). In contrast to Kanchupalli’s work, this reaction underwent LED-irradiated C–H coupling of 2-arylbenzimidazole **60** with iodonium ylide **13** in the presence of Rh(III) and an EosinY co-catalyst at room temperature, leading to a variety of *N*-fused polycyclic compounds **61** in high yields. Excellent functional group tolerance and regioselectivity of this catalytic system were observed.

**Scheme 23:**
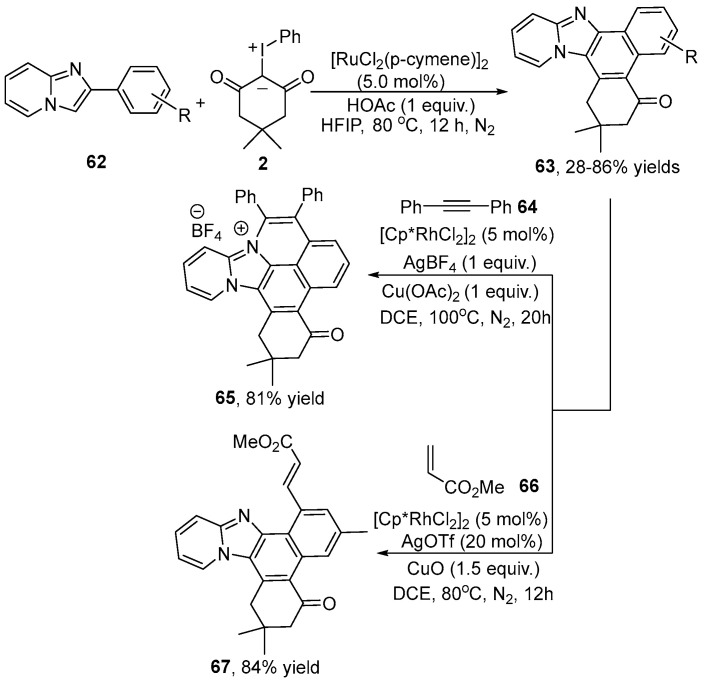
Ru(II)–catalyzed [4+2] cyclization of 2-arylimidazo[1,2-a]pyridines with iodonium ylides.

A Ru(II)-catalyzed C–H bond activation and tandem cyclization of 2-arylimidazo[1,2-*a*]pyridine **62** with iodonium ylide **2** to synthesize pyrido[1,2-*a*]benzimidazole derivative **63** was revealed by Wang and co-workers [38] (Scheme 23). Furthermore, product **63** reacted with 1,2-diphenylethyne and methyl acrylate to form the corresponding coupling compounds **65** and **67** through C–H activation in the present of an Rh(III) catalyst. In addition, a gram-scale (5 mmol) synthesis of pyrido[1,2-*a*]benzimidazole **63** was carried out, achieving a good yield of 67%.

**Scheme 24:**
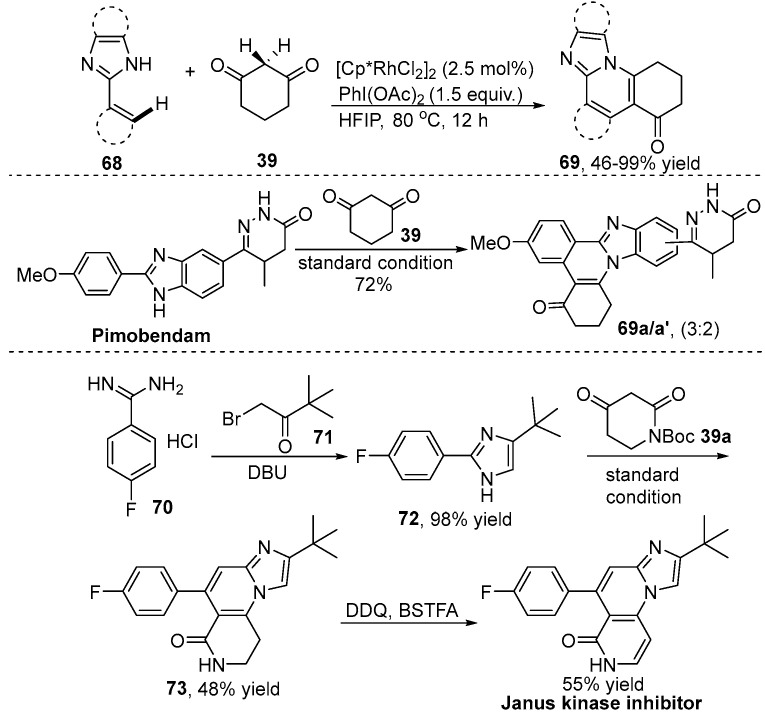
Rh(III)–catalyzed C–H [4+2] cyclization of imidazole with 1,3-dicarbonyl compounds and the total synthesis of a Janus kinase inhibitor.

Arylimidazoles are potential substrates because the imidazole moiety has a strong ability to coordinate with the transition-metal catalyst and serves as a nucleophilic group for intramolecular cyclization. In this respect, Tao and co-workers [39] demonstrated a Rh(III)-catalyzed C–H coupling/cyclization of alkenyl- or arylimidazoles **68** with cyclic 1,3-dicarbonyl compound **39** for the assembly of imidazo-fused polycyclic compound **69** by selectively cleaving two different C–H bonds in a single step (Scheme 24). In this transformation, iodonium ylides were generated in situ from cyclic 1,3-dicarbonyl compound **39** and PhI(OAc)_2_ in one pot. Notably, the imidazole moiety served as a major directing group that played a significant role in controlling the regioselectivity. Excellent functional group compatibility, readily available starting materials and easy operation were observed in this reaction. Importantly, the potential synthetic utility of the coupling product was demonstrated by synthesizing the highly step-economic synthesis of a Janus kinase inhibitor, which was difficult to efficiently achieve by previous methods, in only three steps.

### 2.7. Switchable Group-Directed C–H Annulation

**Scheme 25:**
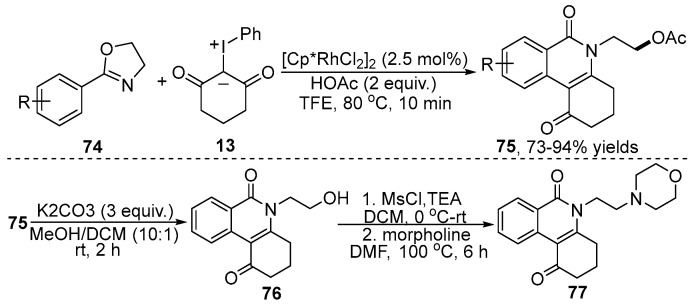
Rh(III)–catalyzed [4+2] annulation of 2-oxazolines with iodonium ylides.

Switchable group-directed C–H functionalization is of great significance for the synthesis of valuable skeleton structures due to the facile transformations from the switchable group. More recently, Liu and co-workers [40] realized oxazoline-directed Rh(III)-catalyzed C–H functionalization of oxazoline **74** with iodonium ylide **13** to give isoquinolone derivative **75** in a relatively high yield (Scheme 25). This reaction was compatible with a range of oxazolines and iodonium ylides. Moreover, the product could be further functionalized for the construction of the corresponding morpholine derivative **77**, which is useful for diverse chemical transformations in organic synthesis.

**Scheme 26:**
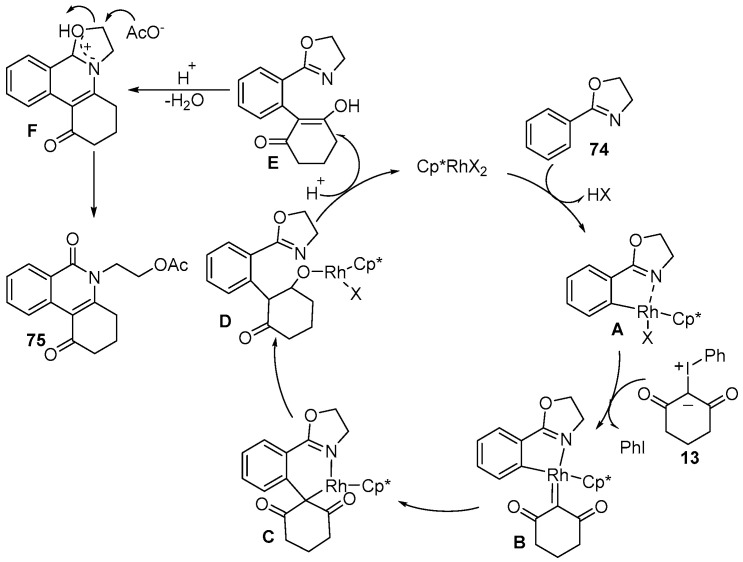
Proposed reaction mechanism for Rh(III)–catalyzed C–H annulation of 2-oxazolines with iodonium ylides.

A possible reaction mechanism is provided in Scheme 26. The reaction was initiated by oxazoline-assisted C–H bond activation to give the five-membered rhodacyclic intermediate **A**. Then, iodonium ylide **13** coordinated with the metal center of intermediate **A** to deliver the active carbene species **B** followed by the elimination of PhI. With subsequent migratory insertion and protonation, the intermediate **E** was generated. The intermediate **E** underwent intramolecular nucleophilic cyclization/dehydration to produce the oxazolinium salt **F**. Finally, acetate attacked the oxazolinium salt **F** to give the expected isoquinolone **75**.

**Scheme 27:**
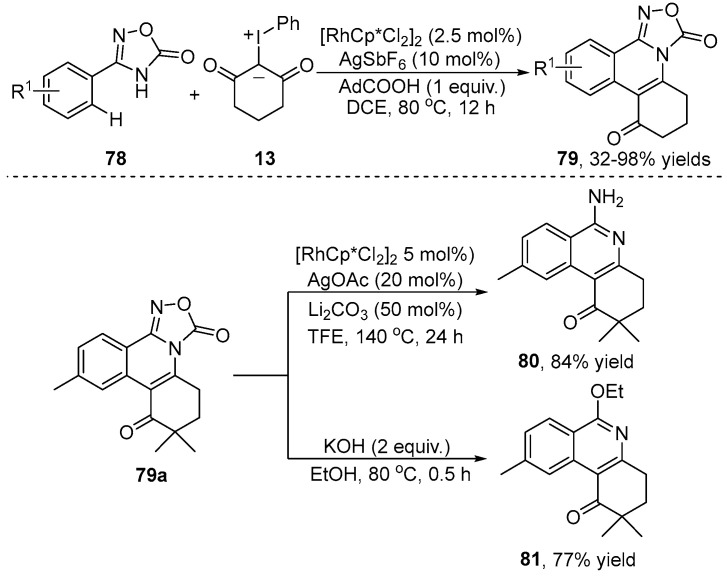
Rh(III)–catalyzed C–H/N–H [4+2] annulation of 3-aryl-1H-indazoles and iodonium ylides.

A Rh(III)-catalyzed C–H/N–H [4+2] annulation between oxadiazolone **78** and iodonium ylide **13** was accomplished by the Shu group [41], which enabled the assembly of fused-isoquinoline **79** (Scheme 27). This protocol showed outstanding functional group tolerance, a wide range of substrates and high atom economy. Furthermore, the authors proved that product **79a** containing oxadiazolones as a switchable directing group could be easily further transformed into the other compounds **80** and **81**. In mechanistic studies, a primary KIE value of 1.7 indicated that the C–H bond cleavage was not involved in the rate-determining step.

**Scheme 28:**
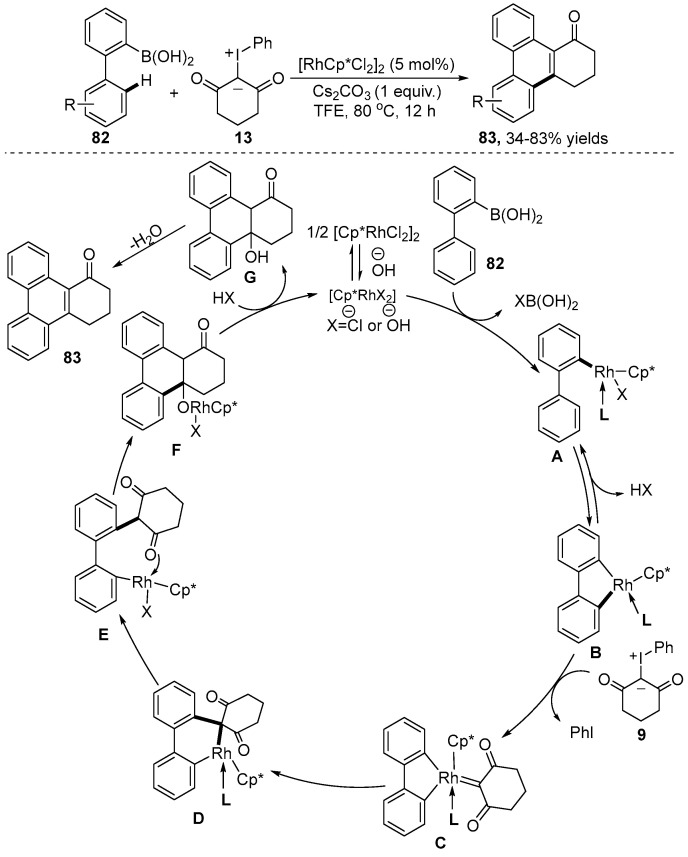
Rh(III)–catalyzed [4+2] annulation of 2-biphenylboronic acid with iodonium ylides.

The Wang group [42] established that transmetalation triggered Rh(III)-catalyzed C–H bond activation and the tandem annulation of 2-biphenylboronic acid **82** with iodonium ylide **13** for the assembly of phenanthrene **83** without any directing group (Scheme 28). The merit of this methodology encompassed stable and easily available substrates, simple operation and redox-neutral conditions. A plausible reaction mechanism was proposed. The reaction was initiated by the transmetalation of substrate **82** with the active catalyst Cp*RhX_2_ to provide intermediate **A**. Subsequently, intermediate **A** underwent intramolecular C–H bond activation, coordination with iodonium ylide **13**, migratory insertion and hydrolysis, to synthesize intermediate **E**. Ultimately, product **83** was obtained by intramolecular nucleophilic cyclization, protonation and intramolecular dehydration.

## 3. Iodonium Ylides Serve as C3 Synthon in C–H Annulation

In comparison to the [n+2] annulation induced by the nucleophilic attack of directing groups against the carbonyl group of iodonium ylides, the [n+3] annulation reaction between the electrophilic directing groups and the nucleophilic enol hydroxyl moiety of iodonium ylides have aroused widespread interest. This method provides a facile and convenient approach to generate multifarious fused heterocyclic compounds.

**Scheme 29:**
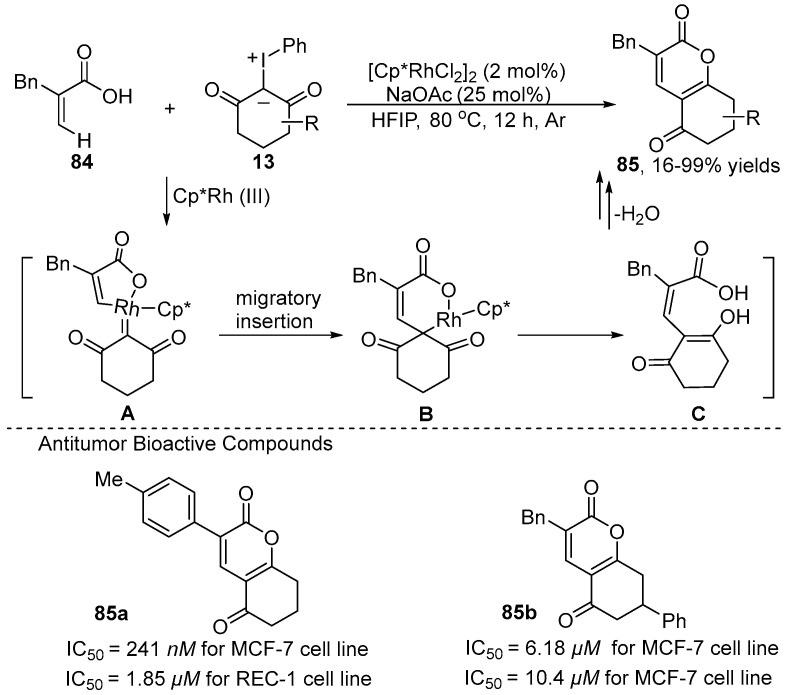
Rh(III)–catalyzed olefinic C–H [3+3] annulation of 2-benzylacrylic acid with iodonium ylides.

The first example of using iodonium ylides as carbene precursors in C–H functionalization was reported in 2020 by Li and co-workers [14] (Scheme 29). They revealed [Cp*RhCl_2_]_2_-catalyzed cross-coupling [3+3] annulation reactions of 2-benzylacrylic acid **84** with iodonium ylide **13** to yield dihydro-2*H*-chromene derivative **85** with high anti-tumor activities. Meanwhile, the catalyst load could be decreased to 0.5 mol% in a gram-scale synthesis, which has great prospects for industrial applications. In this mechanism (Scheme 29), intermediate **A** is generated through C–H activation and coordination with iodonium ylides, which undergoes migratory insertion and protonation to produce intermediate **C**. Then, intermediate **C** undergoes intramolecular nucleophilic cyclization/dehydration to deliver the target product **85.**

**Scheme 30:**
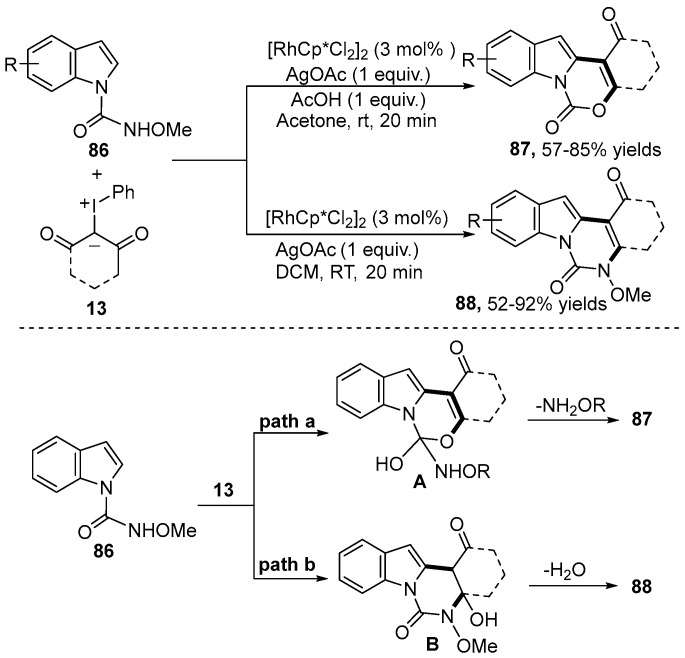
Rh(III)–catalyzed C–H annulation of *N*-carboxamide indoles with iodonium ylides.

Subsequently, a condition-controlled chemodivergent cyclization of *N*-carboxamide indole **86** with iodonium ylide **13** employing [RhCp*Cl_2_]_2_ as a catalyst was reported by the Kanchupalli group [43] (Scheme 30). This methodology provided rapid access to functionalized 1*H*-[1,3]oxazino[3,4-a]indol-1-one derivative **87** and 3,4-dihydroindolo[1,2-*c*]quinazoline-1,6(2*H*,5*H*)-dione **88** under redox-neutral and mild conditions. Note that the acid additives played an important role in the chemoselectivity of this reaction system. The AcOH could activate the amide group that could be attacked by the enol oxygen for the synthesis of the [3+3] annulation product **87** with the elimination of NH_2_OR. On the contrary, the amide NH group could nucleophilically attack the ketone carbonyl of the iodonium ylides to generate the [4+2] annulation product **88** in the presence of AgOAc and DCE.

**Scheme 31:**
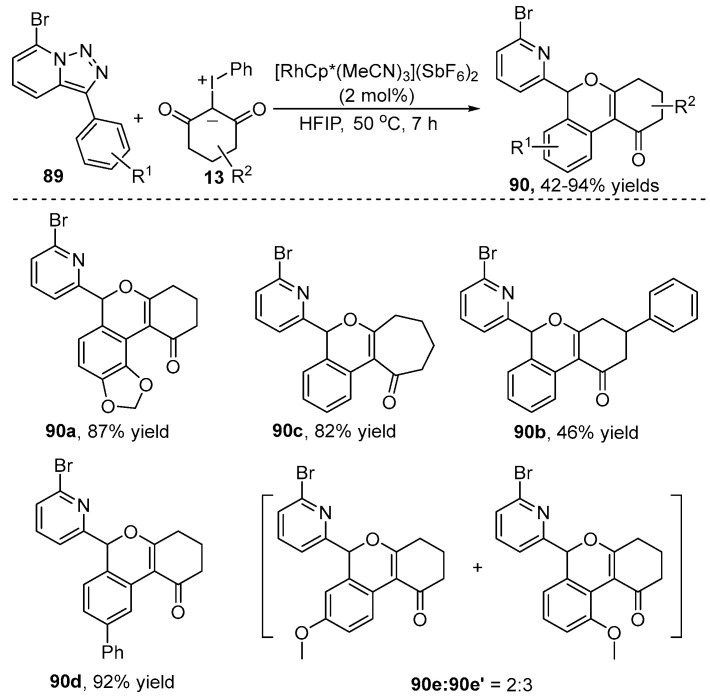
Rh(III)–catalyzed C–H [3+3] annulation of pyridotriazoles with iodonium ylides.

1*H*-isochromene frameworks are ubiquitous in numerous natural products and drugs due to their significant biological activity. A straightforward strategy for the synthesis of 1*H*-isochromene framework **90** without extra additives via a Rh(III)-catalyzed C–H activation/annulation cascade reaction of pyridotriazole **89** with iodonium ylide **13** was disclosed by Wu and co-workers [44] (Scheme 31). In this reaction, a wide range of pyridotriazole **89** and iodonium ylide **13** reacted smoothly to produce the corresponding cyclization products in decent to good yields. However, the meta-substituted substrates with electron-withdrawing groups resulted in mixed products, which indicated the poor regioselectivity in this transformation.

**Scheme 32:**
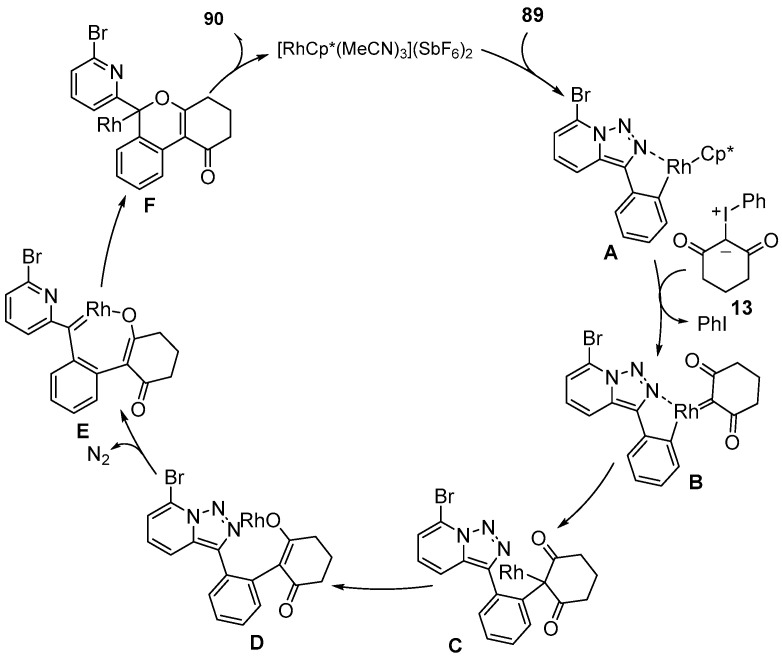
Proposed reaction mechanism for Rh(III)–catalyzed C–H [3+3] annulation of pyridotriazole with iodonium ylides.

A possible mechanism of this reaction is shown in Scheme 32. Firstly, 7-Bromopyrido-triazole **89** coordinated with a [RhCp*(MeCN)_3_](SbF_6_)_2_ catalyst via the C–H activation to generate the five-membered rhodacyclic intermediate **A**, which further coordinated with iodonium ylide **13** to form the rhodium carbene species **B**. Then, intermediate **B** underwent an intramolecular migratory insertion to produce intermediate **C**. Then, a 1,3-shift of intermediate **C** gave the alkoxyrhodium intermediate **D**, which was further converted to the Rh-carbene **E** by denitrogenation. Finally, the intermediate **F** underwent a second migratory insertion and protonation to generate the desired product **90**, followed by the regeneration of the Rh catalyst.

**Scheme 33:**
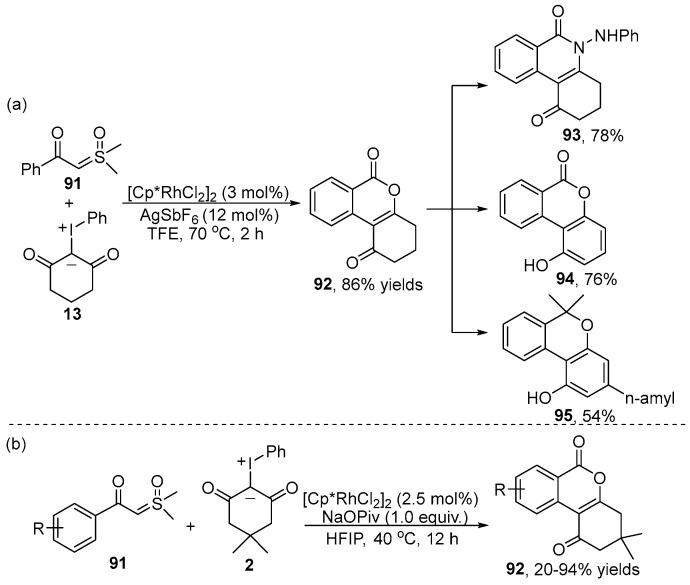
Rh(III)–catalyzed C–H [3+3] annulation of sulfoxonium ylides with iodonium ylides.

Isocoumarin derivatives represent a class of privileged structural motifs with a variety of biological and pharmacological activities, such as anti-fungal, anti-allergic and anticoagulant activities. In 2021, Kanchupalli’s group [45] unveiled a direct strategy to prepare isocoumarin skeleton **92** via the [Cp*RhCl_2_]_2_-catalyzed [3+3] cyclization of sulfoxonium ylide **91** with iodonium ylide **13** using sulfoxonium ylide functionality as the traceless directing group (Scheme 33a). Importantly, to demonstrate the potential application of this annulation reaction, the natural products cannabinol **93**, urolithin core structure **94** and isoquinolone scaffold **95** were rapidly synthesized. Subsequently, a similar Rh(III)-catalyzed synthesis of an isocoumarin skeleton employing the same substrates was developed by Yu and co-workers [46] (Scheme 33b). Excellent functional group tolerance and regioselectivity in this catalytic system were observed.

**Scheme 34:**
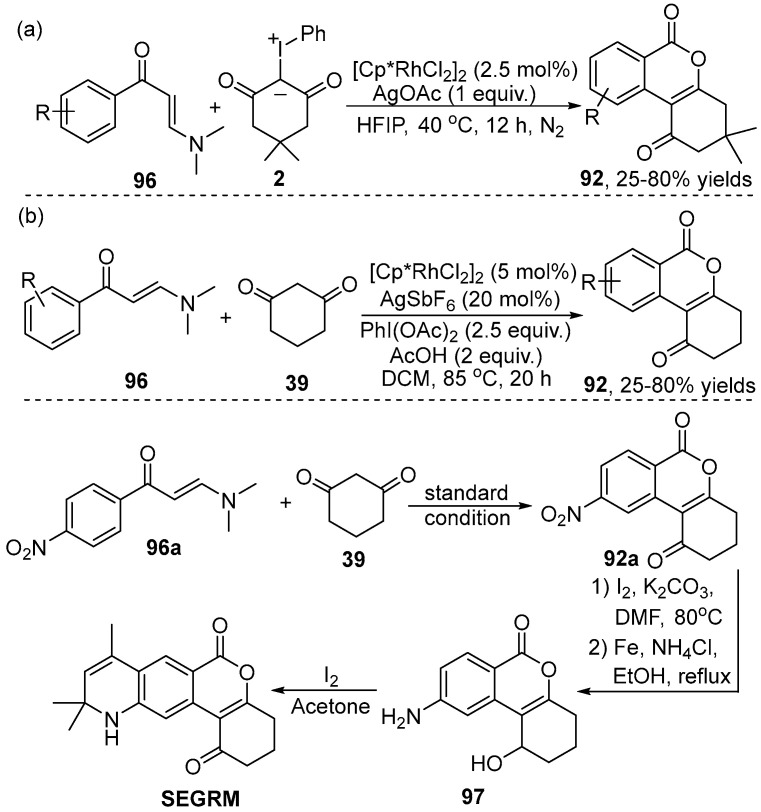
Rh(III)–catalyzed C–H [3+3] cyclization of enaminones and the total synthesis of the nonsteroidal selective glucocorticoid receptor modulator.

Recently, the efficient synthesis of diverse substituted isocoumarin **92** had also been realized by Yu’s group [47] via the Rh(III)-catalyzed cascade annulation reaction of *N*,*N*-dimethyl enaminone **96** with iodonium ylide **2** (Scheme 34a). Additionally, Liu and Li [48] developed a highly simple, efficient one-pot synthesis of isocoumarin **92** through C–H bond activation and intramolecular C–C cascade annulation of enaminone **96** and cyclic 1,3-dicarbonyl **39** by employing [Cp*RhCl_2_]_2_/AgSbF_6_ as co-catalysts, in which the in situ generation of iodonium ylides was perfectly achievable (Scheme 34b). It is worth mentioning that this protocol provided a rapid and efficient method to synthesize the key intermediates of nonsteroidal selective glucocorticoid receptor modulator (**SEGRM**) derivatives with anti-inflammatory properties.

**Scheme 35:**
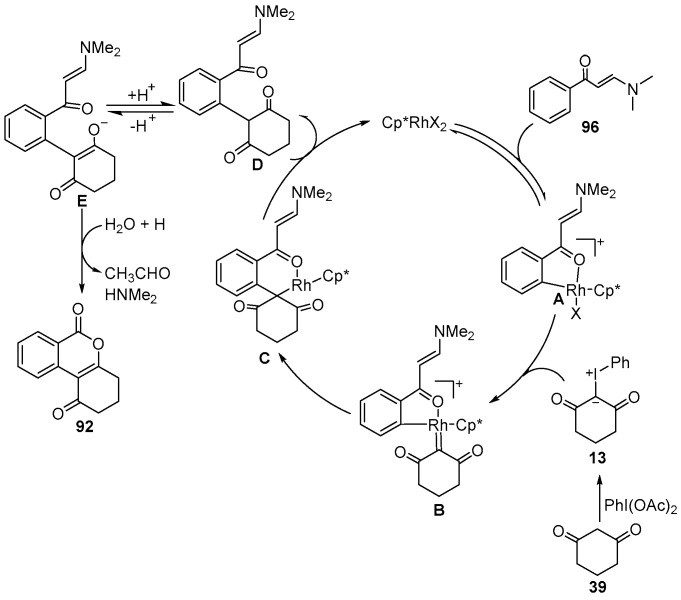
Proposed reaction mechanism for Rh(III)–catalyzed C–H [3+3] cyclization of enaminones with iodonium ylides.

A plausible mechanism is proposed in Scheme 35. Initially, C–H bond activation of enaminone **96** occurred to produce the five-membered rhodacyclic intermediate **A**. Meanwhile, iodonium ylide **13** was formed in situ by the reaction of cyclic 1, 3-dicarbonyl compound **39** with PhI (OAc)_2_. Then, coordination of the iodonium ylide **13** to Rh(III) complex **A** generated a Rh(III) intermediate **B**, which further produced a reactive Rh(III) carbene species **C** via the elimination of PhI. Subsequently, the migratory insertion and protonolysis gave the open-chain alkylation intermediate **D** with the release of the Rh(III) catalyst. Finally, the intermediate **D** was tautomerized to enol **E,** which underwent intramolecular nucleophilic addition and elimination to give the target product **92**.

**Scheme 36:**
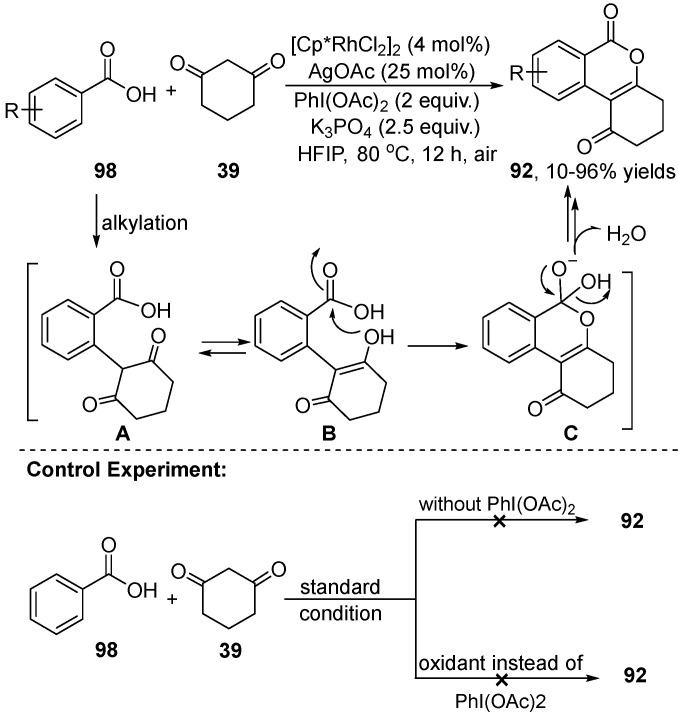
Rh(III)–catalyzed C–H activation of benzoic acids and 1,3-dicarbonyl compounds.

In 2021, Liu [49] also described the synthesis of various isocoumarins **92** by annulation of benzoic acid **98** with the 1,3-dicarbonyl compound **39** through an in situ-generated iodonium ylides process in the presence of PhI(OAc)_2_ (Scheme 36). This reaction was conducted in HFIP using air as an oxidant in the presence of K_3_PO_4_ as a base at 80℃ to generate the products. To elucidate the reaction mechanism, control experiments with benzoic acid **98** and cyclic 1,3-dicarbonyl **39** were performed, and the results demonstrated that iodonium ylides were the key intermediates in the cyclization reaction.

**Scheme 37:**
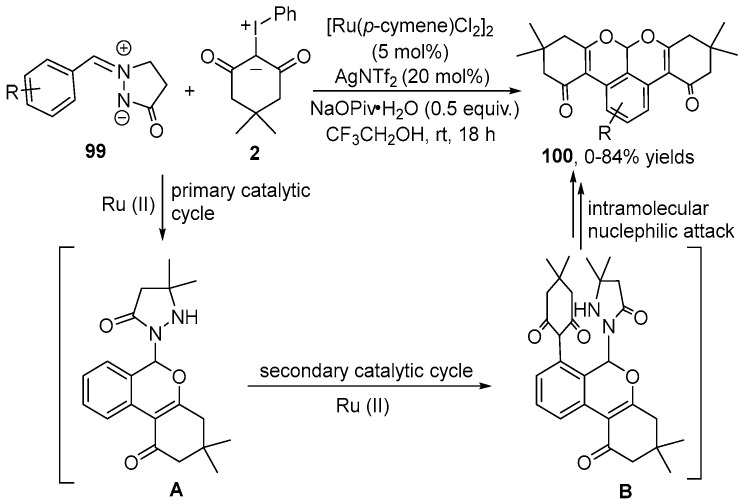
Ru(II)–catalyzed [3+3] annulation of azomethine imines with iodonium ylides.

Given that the Ru(II) catalyst has been shown to efficiently catalyze C–H bond activation reactions with outstanding efficiency, the Wu group [50] described the selective [3+3] cycloaddition of azomethine imine **99** with iodonium ylide **2** using relatively cheap [Ru(p-cymene)Cl_2_]_2_ as the catalyst and the azomethine imine group as a switchable and transient directing group (Scheme 37). This reaction underwent dual C–H bond activation and dual intramolecular nucleophilic attack, leading to a variety of the pyrano[de]isochromene **100**. Furthermore, the H/D exchange experiment indicated that the C–H cleavage was reversible and was faster in the overall reaction. The reaction mechanism was similar to that described above, except for the rearrangement pathway of the directing group in the secondary catalytic cycle.

## 4. Iodonium Ylides Serve as Alkenylating Reagents

**Scheme 38:**
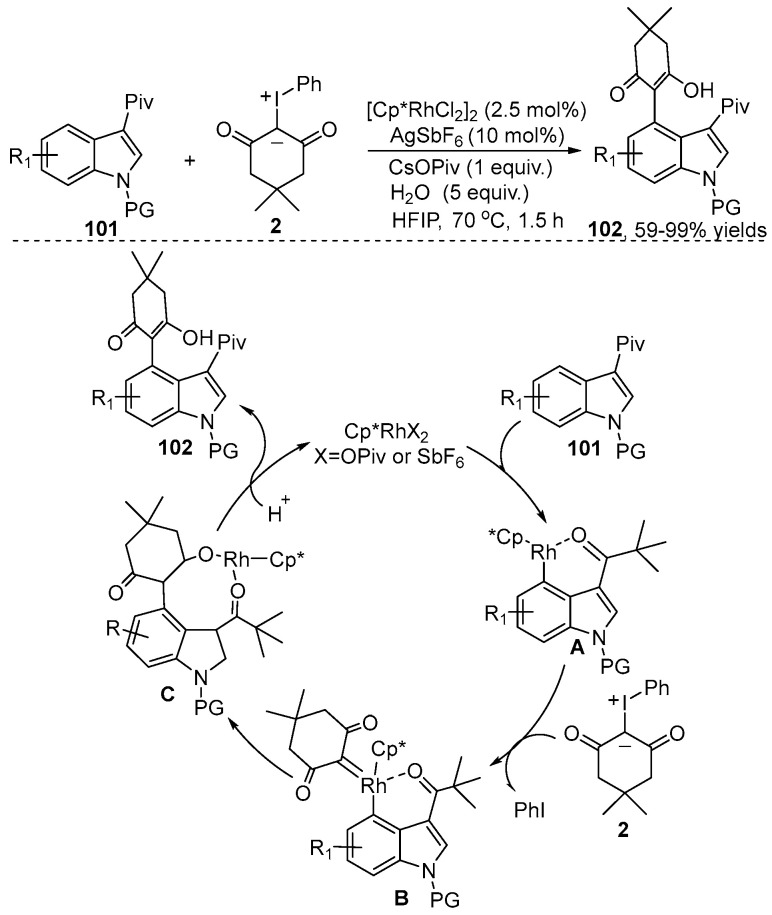
Rh(III)–catalyzed C(4)-selective C–H alkenylation of indoles with iodonium ylides, and the proposed reaction mechanism.

In contrast to the reactions mentioned above, iodonium ylides serve as an effective alkenylating reagent in C–H functionation reactions, which provides an alternative approach to selectively construct the corresponding alkenylating products. In this regard, the Zhang group [51] used pivaloyl as a directing group to accomplish [Cp*RhCl_2_]_2_-catalyzed C4-Selective C–H alkenylation of indole **101** with iodonium ylide **2** as the carbene precursor (Scheme 38). This alkenylation reaction gave product **102** in good yields and a high regioselectivity under redox neutral reaction conditions. A plausible mechanism is proposed (Scheme 38). First, a concerted metalation-deprotonation process of substrate **101** produced a rhodacyclic intermediate **A**. Then, the iodonium ylide **2** coordinated with species **A** to produce the high active rhodium-carbene species **B**, along with the elimination of PhI. Subsequent migratory insertion of the Rh-C bond into the carbene species **B** produced intermediate **C**. Finally, protonation of species **C** delivered the desired product **102** and released the active catalyst.

**Scheme 39:**
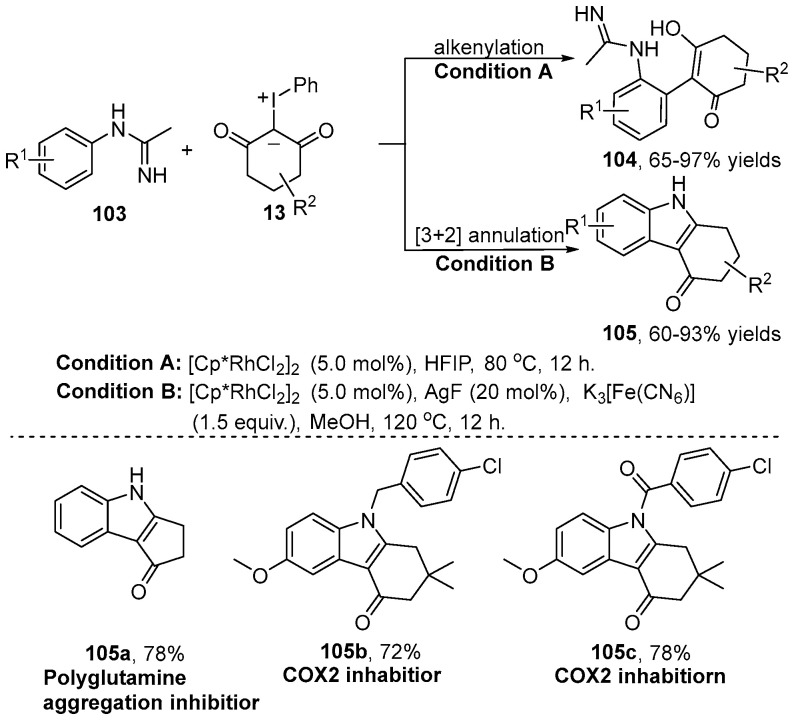
Rh(III)–catalyzed C–H alkenylation and cyclization of *N*-arylamidines with iodonium ylides.

More recently, a condition-controlled reaction of *N*-aryl amidine **103** and iodonium ylide **13** via a Rh(III)-catalyzed C–H bond functionalization had been developed by the Wu group [52] (Scheme 39). This reaction was conducted in HFIP at 80 ℃ to give the alkenylating product **104**. However, switching the solvent to MeOH in the presence of AgF/K_3_[Fe(CN)_6_] as the additive resulted in the formation of the [3+2] product carbazolone **105** through an intermolecular annulation process. It is worth mentioning that this protocol provided rapid and efficient access to synthesize the polyglutamine aggregation inhibitor **105a** and the COX-2 inhibitor **105b–105c**.

**Scheme 40:**
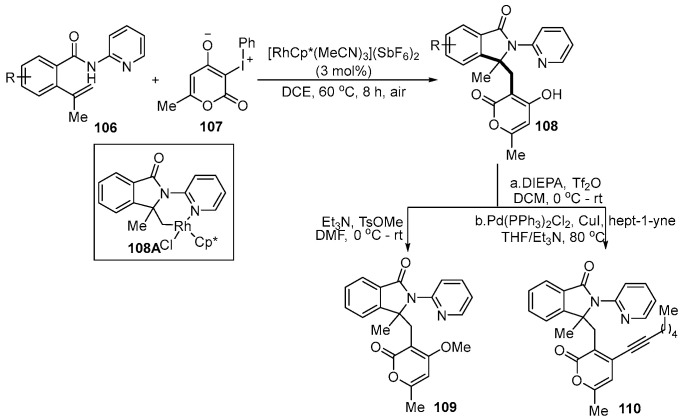
Rh(III)–catalyzed C–H annulation/coupling of isoindolin-1-ones with iodonium ylides.

As we all know, the strategy of molecular hybridization has been widely applied to construct novel and highly-valued organic molecules. In this regard, Mai and co-workers [53] pioneered a Rh(III)-catalyzed tandem coupling reaction of iodonium ylide **107** with the C(sp3)-Rh species, produced by 5-exo-trig cyclization to accomplish molecular hybridization (Scheme 40). To demonstrate the potential application of this annulation reaction, product **108** was further derived to produce the corresponding products **109** and 2-pyrone **110** via methylation and Sonogashira coupling, respectively. Importantly, C(sp^3^)-Rh complex **108A** was proven to be the key intermediate during the mechanism experiment study. The reaction mechanism was proposed to involve metal-mediated 5-exo-trig cyclization, coordination, carbene migration insertion and protonolysis process, in which two new chemical bonds and one stereogenic center were formed at the same time.

**Scheme 41:**
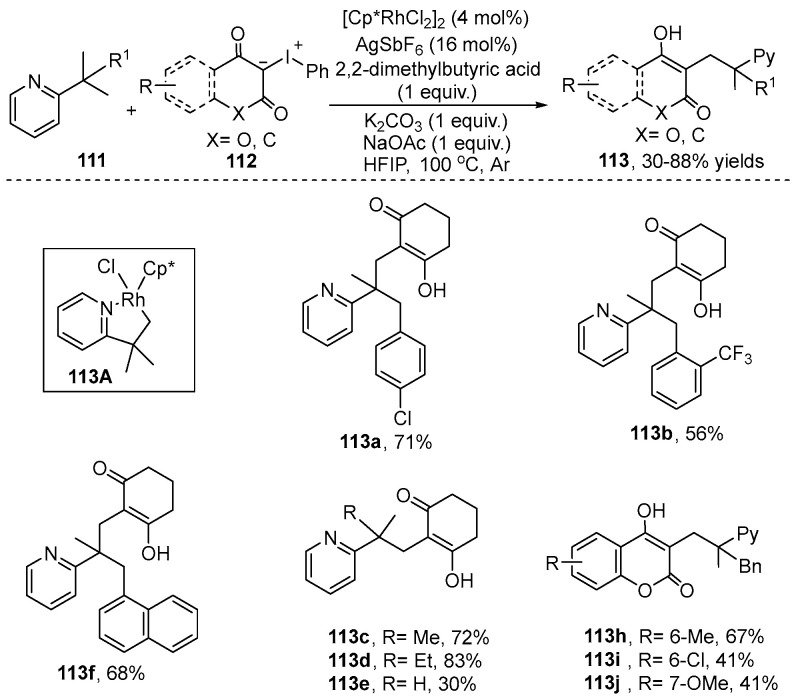
Rh(III)–catalyzed alkenylation of inactivated C(sp3)-H bond with iodonium ylides.

Another C–H alkenylation reaction employing iodonium ylides based on carbene migratory insertion has been demonstrated by Liu and co-workers [54] (Scheme 41). They developed an inactivated methyl C–H alkenylation using iodonium ylide **112** as a carbene precursor to generate the coupling product **113**. A wide range of substrates bearing various functional groups such as halogen, methoxy, trifluoromethyl and naphthyl rings were compatible with this reaction. To explore the reaction mechanism, the stable cyclometalated Rh(III) complex **113A** was isolated, which could efficiently catalyze the reaction to generate the desired product.

## 5. Conclusions

In summary, this paper briefly outlines the application of iodonium ylides in transition-metal-catalyzed C–H activation reactions, including [n+2] cyclization, [n+3] cyclization and alkenylation. Recent research progress indicates that iodonium ylides which serve as a new type of coupling reagent have shown tremendous potential in transition-metal-catalyzed C–H bond activation reactions and have been developed to effectively construct C–C or C–X bonds in complex organic molecules. Although great progress has been made in this field, there are still some limitations and challenges in the application of iodonium ylides. Previous reports have mainly focused on the reaction of iodonium ylides as a C2/C3 synthon and alkenylating reagent, and it is still necessary to expand the reaction model of iodonium ylides, such as potentially in free radical reactions. As for the transition-metal catalysts, Rh(III) and Ru(II) catalysts are the most widely used catalysts, and low-cost metal catalysts and highly active catalytic systems such as Ir, Pd and Co still need to be developed. In addition, the range of iodonium ylide substrates involved in such reactions are mainly confined to cyclic iodonium ylide compounds, while the metal-catalyzed C–H activation reactions with acyclic-derived iodonium ylides have rarely been reported. Therefore, the design and synthesis of iodonium ylides with more diverse structures to allow participation in C–H activation reactions remains a hot topic for future research. Above all, future research will further promote the application and development of iodonium ylides along these lines and bring more innovation and breakthroughs in the field of organic synthesis.

## Data Availability

No new data were created or analyzed in this study. Data sharing is not applicable to this article.
